# Analysis of dynamic contrast-enhanced T1-weighted imaging parameters in type II TIC breast lesions

**DOI:** 10.3389/fonc.2025.1403262

**Published:** 2025-08-12

**Authors:** Jimei Jiang, Weibin Ma, Ming Li, Shanhua Han, Yu Luo

**Affiliations:** Department of Radiology, Shanghai Fourth People’s Hospital, School of Medicine, Tongji University, Shanghai, China

**Keywords:** breast cancer, time signal intensity curve, multivariable analysis, classification, nomogram

## Abstract

**Background:**

In dynamic contrast-enhanced T1-weighted imaging (DCE-T1WI) of breast lesions, type III time–intensity curves (TICs) are associated with malignant lesions, and type I TICs are associated with benign lesions, but the association of type II curves with the status of breast lesions remains controversial. This study aimed to analyze the semi-quantitative parameters derived from DCE-T1WI in patients with type II TIC breast lesions and to develop a nomogram for the benign/malignant classification of lesions with type II TICs.

**Methods:**

Data of patients with type II TIC breast lesions were retrospectively collected. The following semi-quantitative parameters were collected: signal intensity of pre-contrast (SIpre), peak signal intensity (SIp), signal intensity of wash-in (SIwi), slope of peak (Sp), slope of wash-in (Swi), time to peak (Tp), time of wash-in (Twi), enhancement rate of peak (ERp), and enhancement rate of wash-in (ERwi). Univariable and multivariable analyses were performed to select useful clinical and DCE-T1WI features. Selected features were used for nomogram model development. The sensitivity, specificity, positive predictive value (PPV), negative predictive value (NPV), accuracy, and area under the curve (AUC) were used for model performance evaluation.

**Results:**

Ninety-eight female patients with type II TIC breast lesions were included (53 with malignant lesions). After univariable and multivariable logistic regression analyses, only Tp showed an odds ratio of 0.95 (p = 0.014, 95% confidence interval: 0.93–0.97). A nomogram was constructed and included Swi, SIp, SIwi, SIpre, Tp, ERp, and Sp. The sensitivity, specificity, PPV, NPV, and accuracy of the nomogram were 0.827, 0.761, 0.795, 0.796, and 0.796, respectively. The AUC was 0.862.

**Conclusion:**

DCE-T1WI semi-quantitative parameters were different among benign and malignant lesions in patients with type II TIC lesions. Tp showed the most significant difference after a multivariable logistic regression analysis. The results suggest that DCE-T1WI semi-quantitative parameters can be used to predict malignant lesions in patients with type II TIC.

## 1 Introduction

Breast cancer is the most prevalent malignant tumor in women and is a major disease affecting women’s health worldwide (1, 2). Data from the National Central Cancer Registry of China showed that the incidence rate of breast cancer has been increasing in recent years (3), especially in younger women (4). A Chinese cohort showed that the 5-year survival rates were 95.45%, 92.21%, 81.74%, and 67.24% for breast cancer stage I, IIA, IIB, and III-IV, respectively (5). The early detection, early diagnosis, and early treatment of breast cancer can improve the 5-year survival rate and reduce mortality (5–7). Moreover, early detection can provide more favorable conditions for further fertility preservation (8), but overdiagnosis and overtreatment must also be avoided (9). Therefore, establishing a classification model for breast tumor benign or malignant status is of great clinical significance.

Medical imaging techniques provide possibilities for the early detection of breast cancer, and the improvements in early detection rely on the application of advanced imaging equipment, techniques, and software (10, 11). Magnetic resonance imaging (MRI) possesses several advantages among various imaging modalities. Indeed, MRI involves no radiation exposure and can acquire imaging using multiple sequences and from multiple directions, which are unique advantages for breast examination (12–14). MRI also plays an important role in the detection, diagnosis, and prognosis of breast cancer, and MRI results can provide information for surgical plan decisions (15, 16).

Dynamic contrast-enhanced T1-weighted imaging (DCE-T1WI) is an important sequence in breast MRI examination, with superiority in the diagnosis of breast cancer (17, 18). DCE-T1WI can provide the morphological and signal characteristics of breast lesion areas (19). Semi-quantitative and quantitative parameters derived from DCE-MRI can be effective in diagnosing breast cancer, offering high sensitivity for detecting malignant lesions, but limitations include variability between studies, dependence on imaging protocol, and potential for false positives due to factors like breast density and the need for careful interpretation alongside clinical information. Quantitative parameters may provide more precise information about tumor characteristics compared to semi-quantitative assessments, but longer scan times can limit clinical applicability (20–23).

After region of interest (ROI) selection, the software can generate a time–intensity curve (TIC) for each lesion. TIC can reflect the blood supply characteristics in the ROI. Malignant lesions display neovascularization, and neovascular endothelial cells are immature with high permeability, resulting in a rapid enhancement after dynamic enhancement phases; however, benign lesions have relatively less blood supply and often show milder enhancement (24). The different TIC patterns provide great values for the differential diagnosis of breast cancer (25). TIC is further divided into three standard types in breast cancer imaging based on their morphology in the delayed phase (post-peak, typically after the initial 2 minutes following contrast injection): type I (persistent rise), characterized by a continued increase in signal intensity after the initial uptake, shows high specificity for benign lesions; type III (washout), defined by a decrease in signal intensity after reaching an initial peak, shows high specificity for malignant lesions; and type II (plateau), where the signal intensity reaches a peak in the initial phase (within the first 2 minutes) and then flattens (remaining within ±10% of the peak value) during the delayed phase, for which the association with breast tumor benign or malignant classification is controversial (26). Previous studies have shown that type II enhancement curves have a specificity of approximately 72%–75% for the diagnosis of malignant breast cancer (27–29). These results emphasize the difficulty in the differential diagnosis of benign and malignant breast lesions in type II TIC patients.

Therefore, it is clinically important to establish a prediction model based on DCE-T1WI TICs and clinical information to distinguish benign from malignant breast lesions.

This study retrospectively analyzed the relationship between type II TIC lesions and semi-quantitative DCE-T1WI parameters.

## 2 Methods

### 2.1 Study design and patients

This retrospective study included patients with type II TIC breast lesions who underwent preoperative MRI and breast lesion resection in the Department of Breast Surgery between January 2019 and July 2022.

The inclusion criteria were 1) patients with breast dynamic enhancement MRI examination in the study hospital; 2) complete medical information; 3) imaging diagnosis of type II TIC breast lesion; 4) no needle biopsy, radiotherapy, or chemotherapy before the MRI examination; and 5) available postoperative pathological examination. The patients with poor imaging quality were excluded, e.g., motion artifacts due to the inability of the patients to remain still or metal artifacts due to chest or upper arm skin tattoos with inks containing minerals and metals.

This study was approved by the ethics committee of Shanghai Fourth People’s Hospital (approval ID: 2021-047-001). The requirement for individual informed consent was waived by the committee due to the retrospective nature of the study.

### 2.2 MRI acquisition

All examinations were performed using a 3.0 T MR scanner (Prisma, Siemens, Erlangen, Germany) with a standard 18-channel dedicated bilateral breast coil. Breast MR protocol included routine non-contrast T1W, T2-weighted (T2W), diffusion-weighted imaging (DWI), and DCE-T1WI. DWI was acquired using a single-shot echo planar imaging (EPI) sequence on the transverse plane. The b-values were set to 0 or 1,000 s/mm^2^. The apparent diffusion coefficient (ADC) mapping, along with slice selection, phase encoding, and frequency encoding directions, was computed on the workstation. DCE-MRI was acquired using a fast low-angle shot (FLASH) sequence at the transverse plane with the number of excitations set to 1. The MR plain scan was first acquired before contrast injection. Then, the contrast [gadopentetate dimeglumine (Gd-DTPA)] was injected into the cubital vein at 2.5 mL/s, and the residual contrast agent in the tube was flushed with 20 mL of normal saline. Meanwhile, the DCE scanning program was started. The DCE scanning was repeated five times, with each round lasting approximately 1 minute.

### 2.3 Image analysis

The images were transferred to the picture archiving and communication system (PACS; United-Imaging Healthcare, Wuhan, China). Multiple semi-quantitative parameters were collected, including signal intensity of pre-contrast (SIpre), peak signal intensity (SIp), signal intensity of wash-in (SIwi), slope of peak (Sp), slope of wash-in (Swi), time to peak (Tp), time of wash-in (Twi), enhancement rate of peak (ERp), and enhancement rate of wash-in (ERwi). SIpre was the SI before enhancement. SIp was the peak SI in the whole period. Tp was the duration between contrast injection and the SI of the ROI reaching SIp. SIwi was the maximal SI in the wash-in period. Twi was the duration between contrast injection and the SI of the ROI reaching SIwi. Sp, ERp, Swi, and ERwi were calculated according to the following equations.


Sp=(SIp−SIpre)/Tp,

Swi=(SIwi−SIpre)/Twi,

ERp=(SIp−SIpre)/SIpre×100%,

ERwi=(SIwi−SIpre)/SIpre×100%.

DCE-T1WI images were transferred to the Siemens post-processing workstation (mean curve: Siemens, Erlangen, Germany) to obtain the TIC. Post-processing was performed by two senior radiologists with >15 years of experience in breast cancer MRI, both blinded to the pathological diagnosis. Round ROIs for TIC were placed independently by the two radiologists, and they were as large as possible and covered the most prominent area of enhancement in the lesion. The size of the ROI had to be >0.3 cm^2^. ROI delineation had to avoid obvious necrosis, hemorrhage, and cystic changes. The software automatically generated the TIC. In case of disagreement, the final ROI selection and radiological diagnosis were made after discussion. Three time points (time point of contrast injection, Twi, and Tp) were selected, and four parameter mappings [wash-in, washout, maximum intensity projection over time (MIPt), and positive enhancement integral (PEI)] of breast perfusion pseudo-color images were generated. The signals of the whole lesion, the peripheral and central parts of the lesion, and the opposite side of the normal breast were documented.

### 2.4 Pathological analysis

Pathological diagnosis was taken as the reference in this study. The levels of estrogen receptor (ER), progesterone receptor (PR), C-erbB2, Ki67, CK7, SMMS-1, P63, and E-cad index were determined by immunohistochemical examination (IHC) of the resected breast cancer specimens.

### 2.5 Statistical analysis

Stata 17.0 (Stata Corporation, College Station, TX, USA) was used in this study. The continuous variables were tested for the normality of their distribution using the Shapiro–Wilk test. Normally distributed continuous variables were presented as means ± standard deviations (SDs) and analyzed using Student’s t-test (two groups) or ANOVA (three or more groups). Non-normally distributed continuous variables were presented as medians (lower quartiles, upper quartiles) and analyzed using the Kruskal–Wallis H-test (two groups) or ANOVA (three or more groups).

Univariable and multivariable Cox regression analyses were performed to investigate the association between various DCE-T1WI features and the benign/malignant classification of breast lesions. Variables with a p< 0.05 in the univariable analysis were included in the multivariable analysis and nomogram model development. The performance of the nomogram was validated using sensitivity, specificity, positive predictive value (PPV), negative predictive value (NPV), accuracy, and area under the receiver operating characteristic curve (AUROC). A decision curve analysis (DCA) was performed. In this study, p-values were retained to three decimal places, and p< 0.05 was considered statistically significant.

## 3 Results

### 3.1 Characteristics of the patients

Ninety-eight patients with type II TIC lesions were included in this retrospective study. Their median age was 54 years; all patients were female (100%), 25.5% had hypertension, 11.2% had diabetes, and 17.4% had hyperlipidemia. **Table 1** shows the clinical and DCE-T1WI characteristics of the patients. Age was significantly different between the benign and malignant groups (40.0 vs. 64.0 years, p< 0.001), as well as boundary enhancement (13.3% vs. 43.4%, p = 0.001) and ADC (1.31 ± 0.14 vs. 0.86 ± 0.18 × 10^−3^, p< 0.001). For semi-quantitative DCE-T1WI parameters, seven of nine features showed significant differences between the benign and malignant groups (all p< 0.05); only SIp and Twi were not significantly different (p = 0.131 and p = 0.135, respectively).

**Table 1:** Clinical information and DCE-MRI parameters in benign and malignant groups.

Parameters	All (n = 98)	Benign (n = 45)	Malignant (n = 53)	p^#^
Age	53.50 (40.00, 66.00)	40.00 (34.00, 47.75)	64.00 (54.75, 68.25)	**<0.001**
Hypertension, n (%)	25 (25.5)	10 (22.2)	15 (28.3)	0.491
Diabetes mellitus, n (%)	11 (11.2)	3 (6.7)	8 (15.1)	0.188
Hyperlipidemia, n (%)	17 (17.4)	6 (13.3)	11 (20.8)	0.334
Tumor enhancement type				0.498
Non-mass, n (%)	96 (98.0)	45 (100)	51 (96.2)	
Mass-like, n (%)	2 (2.0)	0	2 (3.8)	
Boundary enhancement, n (%)	29 (29.6)	6 (13.3)	23 (43.4)	**0.001**
Initial enhancement, n (%)	98 (100)	45 (100)	53 (100)	--
ADC value (×10^−3^)^*^		1.31 ± 0.14	0.86 ± 0.18	**<0.001**
SIpre	119.15 (99.30, 190.48)	109.40 (91.72, 129.85)	134.45 (105.08, 266.60)	**0.003**
SIp	473.00 (384.93, 586.20)	461.05 (377.07, 534.32)	492.85 (387.25, 685.80)	0.131
Tp	120.00 (71.00, 120.00)	120.00 (120.00, 120.00)	73.00 (71.00, 120.00)	**<0.001**
Sp	3.76 (2.86, 4.57)	3.05 (2.46, 3.85)	4.23 (3.49, 5.21)	**<0.001**
ERp	2.58 (1.82, 3.19)	3.01 (2.40, 3.42)	2.27 (1.54, 2.84)	**0.001**
SIwi	512.60 (420.10, 646.33)	478.85 (403.98, 581.23)	546.75 (451.57, 714.22)	**0.005**
Twi	167.50 (120.00, 217.00)	170.00 (120.00, 219.25)	166.00 (120.00, 170.50)	0.135
Swi	2.48 (1.80, 3.09)	2.17 (1.79, 2.81)	2.71 (2.05, 3.42)	**0.008**
ERwi	2.92 (2.03, 3.53)	3.10 (2.48, 3.79)	2.60 (1.91, 3.40)	**0.047**

Values are median (IQR) or n (%). Bold indicates p < 0.05.

ADC, apparent diffusion coefficient; TIC, time–intensity curve; SIpre, signal intensity of pre-contrast; Tp, time to peak; SIp, peak signal intensity; Sp, slope of peak; ERp, enhancement rate of peak; Twi, time of wash-in; SIwi, signal intensity of wash-in; Swi, slope of wash-in; ERwi, enhancement rate of wash-in; DCE-MRI, dynamic contrast-enhanced magnetic resonance imaging; IQR, Interquartile Range.

^*^ Mean ± standard deviation (SD).

^#^ p-Values between benign and malignant groups.

**Figure 1:**
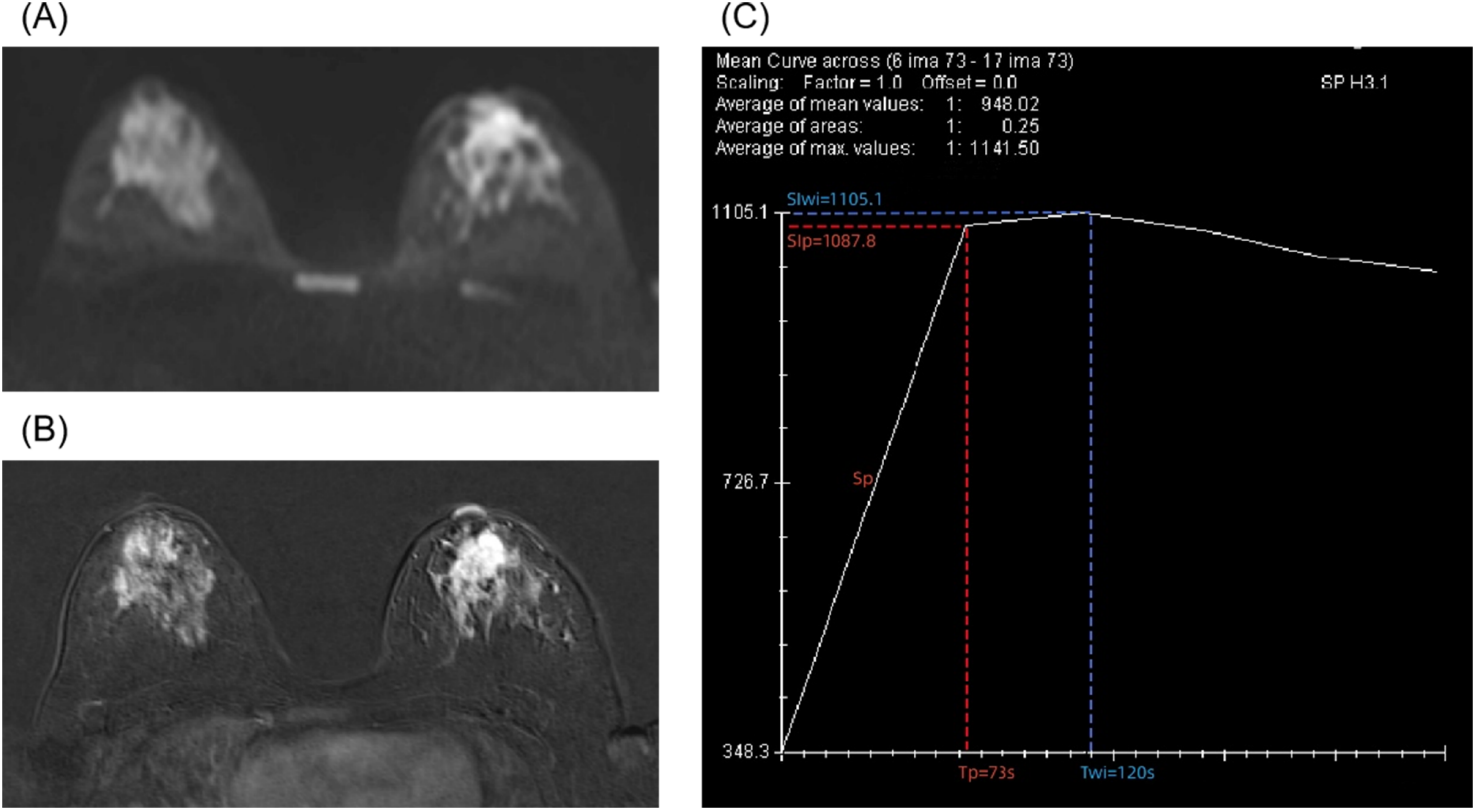
A 28-year-old female patient with grade III invasive ductal carcinoma of the left breast. **(A)** Diffusion-weighted imaging shows a high signal mass with an apparent diffusion coefficient (ADC) of 0.9 × 10^−3^ mm^2^/s. **(B)** Enhanced T1-weighted imaging shows irregular, non-enhanced necrotic area with lobulated signs at the margins. **(C)** A type II time–intensity curve (TIC) is shown. The semi-quantitative parameters are signal intensity of pre-contrast (SIpre) = 348.3, time to peak (Tp) = 73 s, peak signal intensity (SIp) = 1,087.8, slope of peak (Sp) = 10.13, enhancement rate of peak (ERp) = 2.12, time of wash-in (Twi) = 120 s, signal intensity of wash-in (SIwi) = 1,105.1, slope of wash-in (Swi) = 6.31, and enhancement rate of wash-in (ERwi) = 2.17.

**Table 2** shows the clinical and DCE-T1WI features of the patients with different classes. The ERp was lower in grade III lesions compared with grade I–II lesions (median, 1.99 vs. 2.46, p = 0.020), without significant differences for the other parameters. **Figure 1** shows a classic case of female patients with grade III invasive ductal carcinoma of the left breast.

**Table 2:** DCE-MRI parameters in histologic grade I–II and grade III groups among the 53 patients with malignant breast lesions.

Parameters	Histologic grade I–II (N = 31)	Histologic grade III (N = 22)	p
Age	65.00 (57.50, 69.00)	61.50 (53.25, 67.75)	0.320
DCE-MRI parameters			
SIpre	125.70 (104.75, 228.55)	169.60 (109.60, 267.80)	0.260
SIp	487.50 (400.20, 700.50)	526.35 (385.95, 659.68)	0.960
Tp	72.00 (71.00, 120.00)	73.00 (71.00, 73.75)	0.490
Sp	4.49 (3.54, 5.21)	3.96 (3.47, 5.47)	
ERp	2.46 (1.93, 3.04)	1.99 (1.44, 2.41)	**0.020**
SIwi	534.40 (451.05, 718.15)	635.85 (461.62, 696.50)	0.560
Twi	120.00 (120.00, 170.00)	167.00 (120.00, 220.00)	0.150
Swi	2.78 (2.31, 3.45)	2.53 (1.72, 3.25)	0.310
ERwi	2.80 (2.19, 3.44)	2.14 (1.88, 3.03)	0.190
Curve type			0.110
a	18 (58.06)	15 (68.18)	
b	13 (41.94)	7 (31.82)	
Lymph node metastasis			0.705
Yes	4 (12.90)	4 (18.18)	
No	27 (87.10)	18 (81.82)	

Values are median (IQR) or n (%). Bold indicates p < 0.05.

DCE-MRI, dynamic contrast-enhanced magnetic resonance imaging; TIC, time–intensity curve; SIpre, signal intensity of pre-contrast; Tp, time to peak; SIp, peak signal intensity; Sp, slope of peak; ERp, enhancement rate of peak; Twi, time of wash-in; SIwi, signal intensity of wash-in; Swi, slope of wash-in; ERwi, enhancement rate of wash-in.

### 3.2 Univariable and multivariable analyses

Ten features were included in the univariable analyses (**Table 3**). All peak-related DCE-T1WI features exhibited associations with breast lesion malignancy, with p = 0.031, p< 0.001, p< 0.001, and p = 0.022 for SIp, Tp, Sp, and ERp, respectively. However, for the wash-in-related features, Twi and ERwi were not associated with malignancy (p = 0.238 and p = 0.182), and the type of TIC was not associated either (p = 0.374).

**Table 3:** Univariable and multivariable logistic regression analyses of DCE-MRI parameters.

Parameters	Univariable		Multivariable	
DCE-MRI parameters	OR	p	OR (95% CI)	p
SIpre	1.01 (1.00–1.01)	**0.007**	0.98 (0.95–1.02)	0.404
SIp	1.00 (1.00–1.01)	**0.031**	1.01 (0.98–1.05)	0.364
Tp, s	0.95 (0.93–0.97)	**<0.001**	0.90 (0.82–0.98)	**0.014**
Sp	2.54 (1.62–3.98)	**<0.001**	0.25 (0.03–1.74)	0.160
ERp	0.61 (0.40–0.93)	**0.022**	0.82 (0.20–3.47)	0.792
SIwi	1.00 (1.00–1.01)	**0.004**	1.00 (0.99–1.02)	0.614
Twi, s	1.00 (0.99–1.00)	0.238		
Swi	2.03 (1.25–3.31)	**0.004**	1.11 (0.42–2.93)	0.832
ERwi	0.77 (0.52–1.13)	0.182		
Curve type	1.44 (0.64–3.22)	0.374		

Bold indicates p < 0.05.

DCE-MRI, dynamic contrast-enhanced magnetic resonance imaging; OR, odds ratio; CI, confidence interval; TIC, time–intensity curve; SIpre, signal intensity of pre-contrast; Tp, time to peak; SIp, peak signal intensity; Sp, slope of peak; ERp, enhancement rate of peak; Twi, time of wash-in; SIwi, signal intensity of wash-in; Swi, slope of wash-in; ERwi, enhancement rate of wash-in.

**Figure 2:**
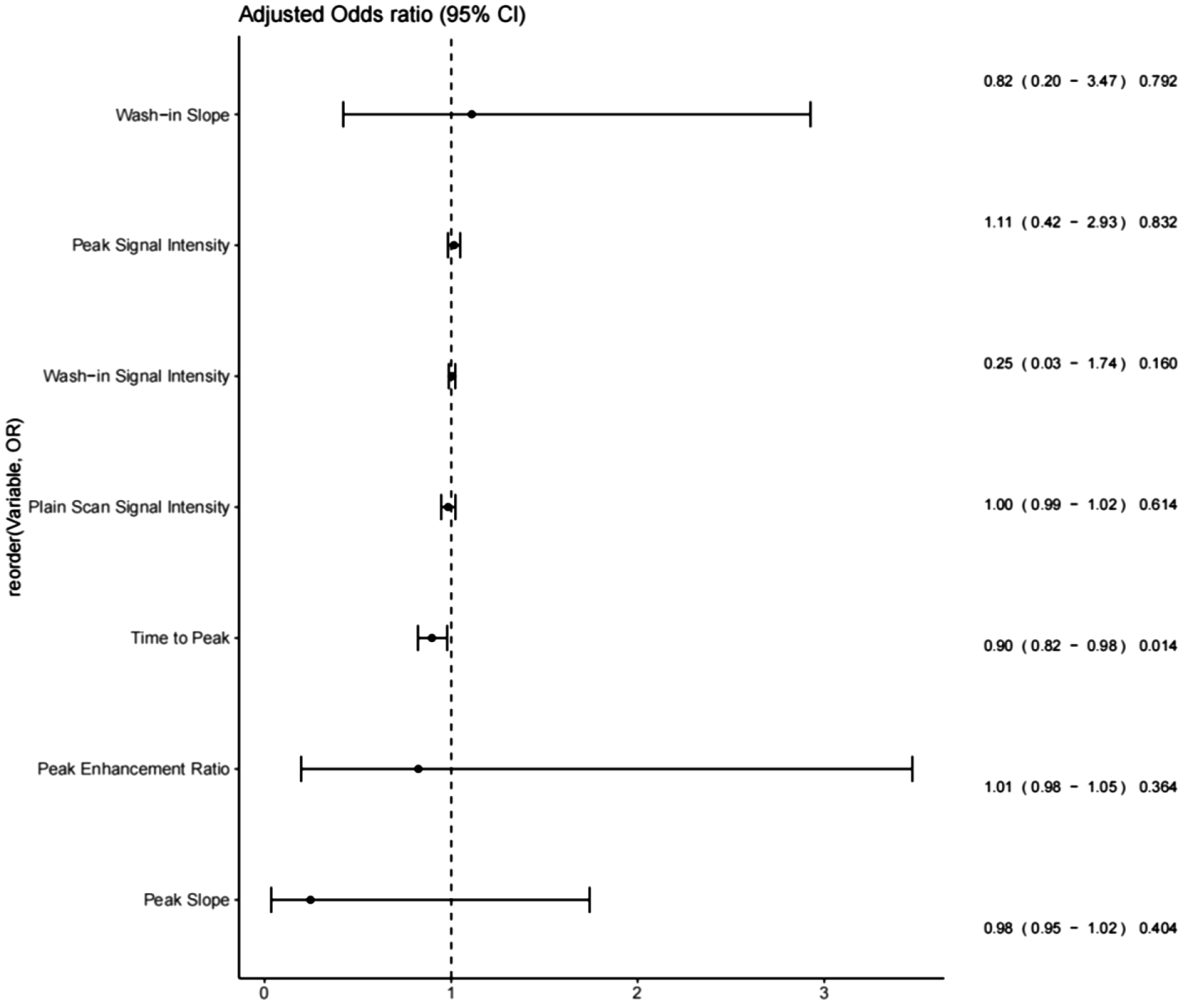
Forest plots of features that survived single-variable analysis.

After the univariable analysis, seven DCE-T1WI features, namely, SIpre, SIp, Tp, Sp, ERp, SIwi, and Swi (all with p< 0.05), were included in the multivariable analysis. Only Tp was statistically significant in the multivariable analysis, with an odds ratio of 0.95 [95% confidence interval (CI) of 0.93 to 0.97, p = 0.014], exhibiting a negative association with malignancy (**Figure 2**, **Table 2**).

### 3.3 Nomogram

**Figure 3:**
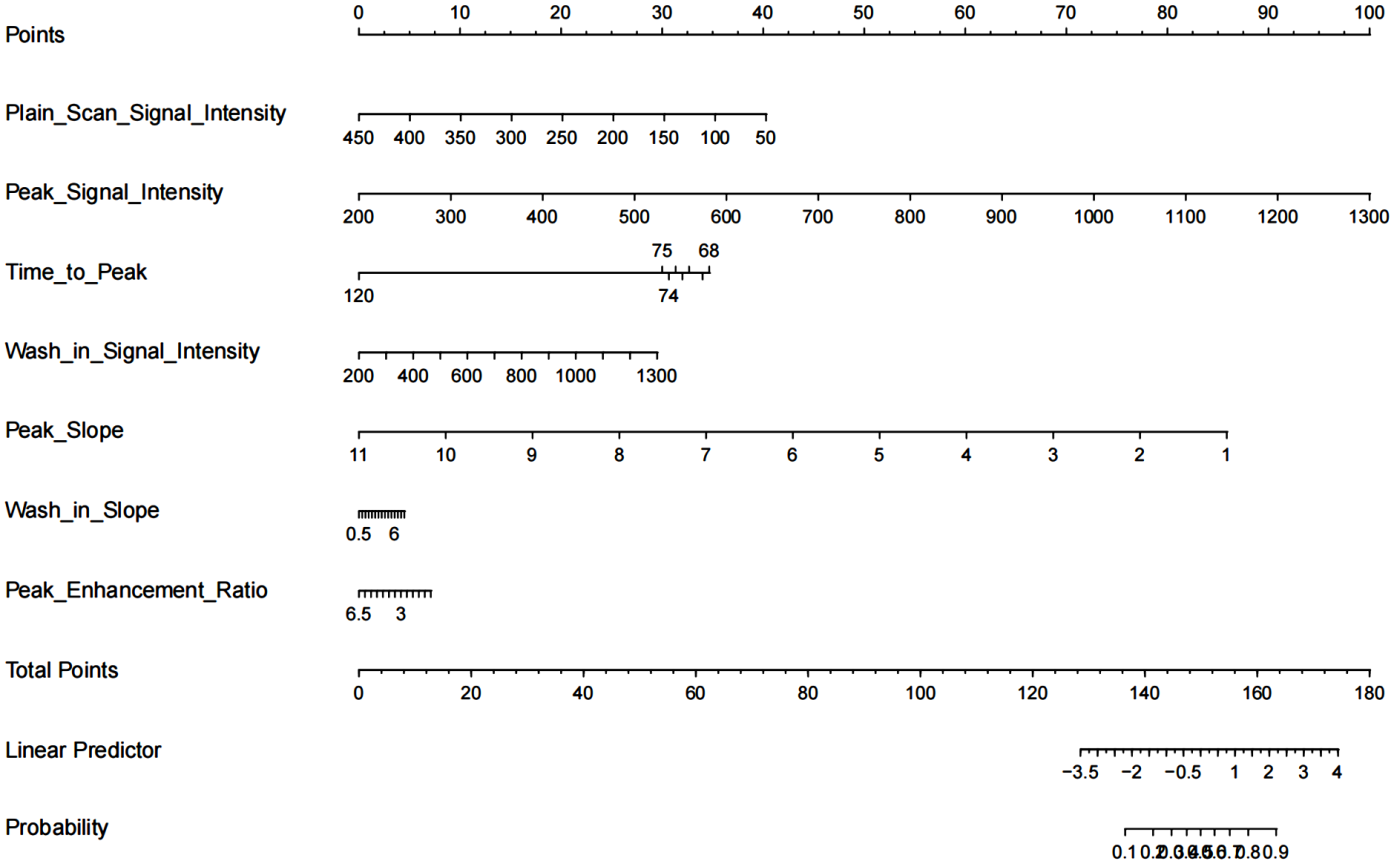
Nomogram for differentiation of benign or malignant lesions with type II time–intensity curve.

**Figure 4:**
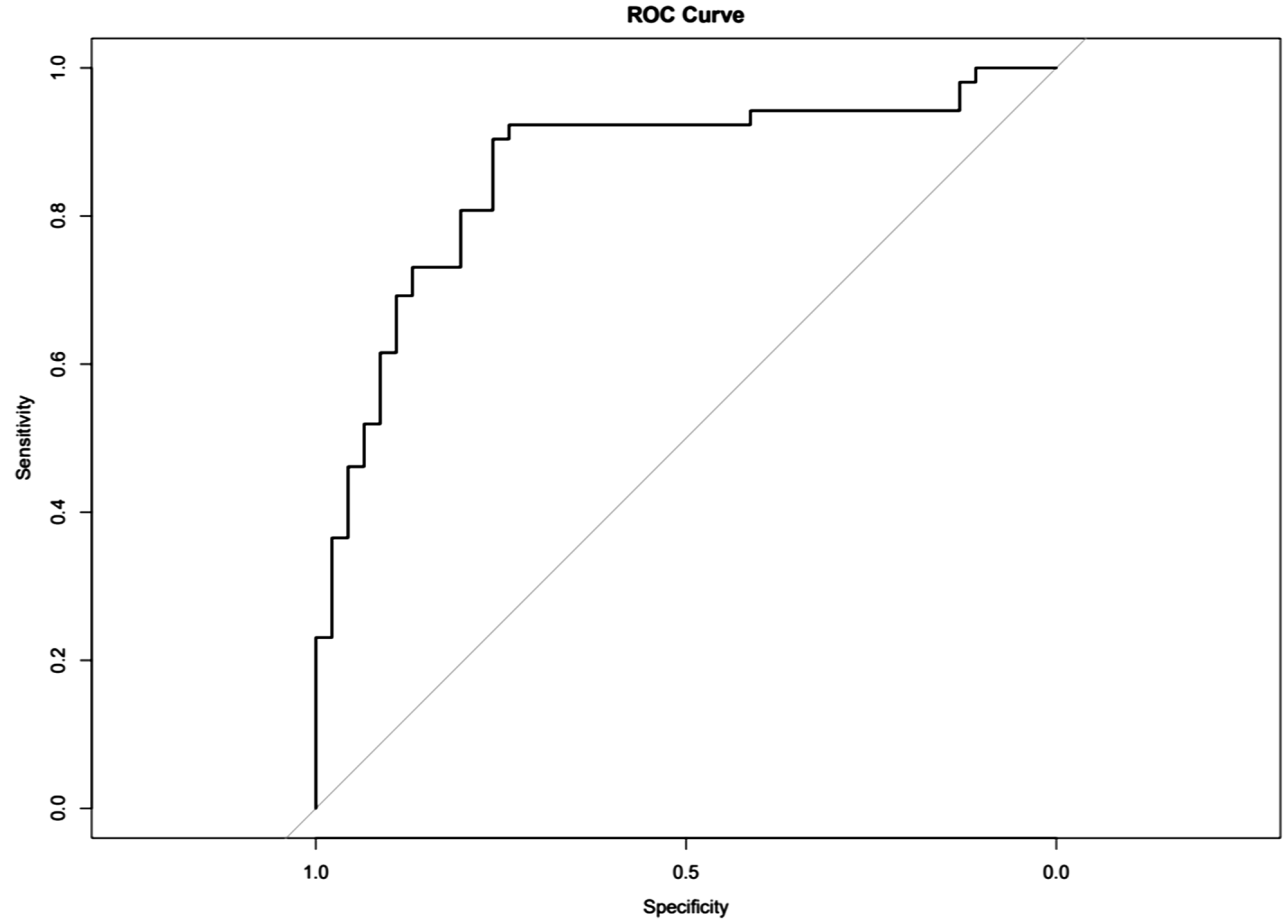
Receiver operating characteristics (ROCs) for differentiation of benign or malignant lesions with type II time–intensity curve.

**Figure 5:**
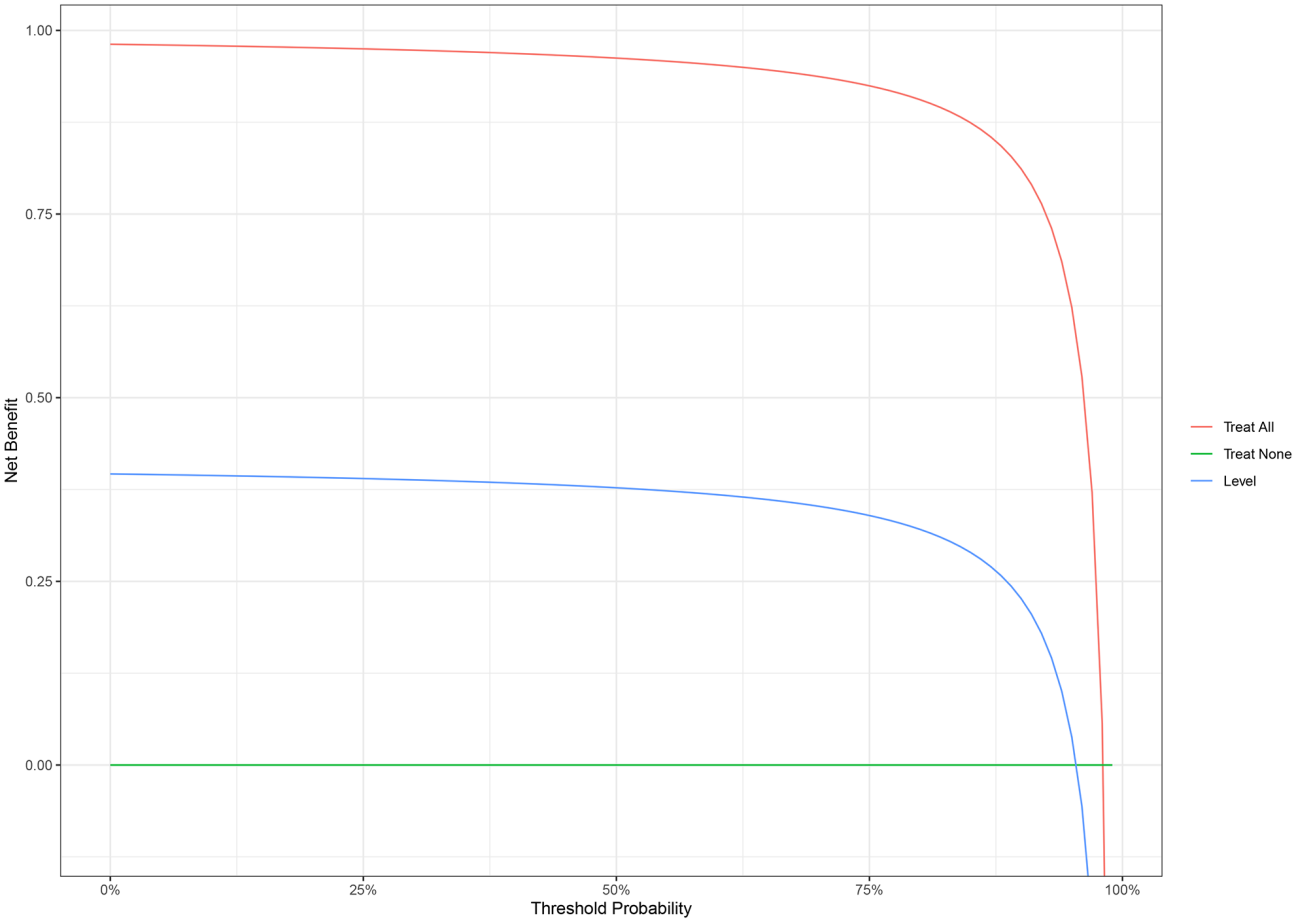
Decision curve analysis (DCA) of the nomogram for malignant breast lesions.

The nomogram with the selected features is shown in **Figure 3**. The sensitivity, specificity, PPV, NPV, and accuracy of the nomogram were 0.827, 0.761, 0.795, 0.796, and 0.796, respectively. The ROC of the nomogram is shown in **Figure 4**. The AUROC was 0.862. The DCA is shown in **Figure 5**.

## 4 Discussion

Indeed, the precise diagnosis of malignant breast lesions in patients with type II TIC lesions has long been a challenging task in clinical management. Many studies have sought to improve diagnostic accuracy by incorporating additional sequences like DWI, demonstrating that parameters like ADC are valuable (30–32). Although several studies have examined the value of DCE-MRI to discriminate between benign and malignant breast lesions, to the best of the authors’ knowledge, this is the first study to incorporate semi-quantitative DCE-T1WI parameters specifically for type II TIC breast lesions, offering a potentially more streamlined diagnostic approach.

Tp was the only factor independently associated with malignant lesions in the multivariable analysis. Tp, defined as the duration between contrast injection and the signal intensity of the region of interest reaching its peak, was significantly shorter in malignant lesions. A shorter Tp seen in malignant lesions could be related to the neovascularization often seen in malignant lesions, leading to higher lesion perfusion and a shorter time to peak. Reynolds et al. showed that Tp was the most discriminating parameter for low-grade prostate tumors, and the performance of Tp was better than the performance of the ADC value (33). Manganaro et al. showed the clinical value of Tp in testicular tumors, finding that Tp was shorter in benign lesions than malignant tumors (34). The results of Tp in testicular malignant tumors were different from those of malignant breast lesions in the present study, suggesting pathological differences between these two cancers. Sp was another feature significant in the univariable analysis. According to Ohashi et al., the maximal slope (MS) showed high stability in triple-negative breast cancer (TNBC), with an intraclass correlation coefficient (ICC) of 0.95, and was associated with TNBC (35). In addition, Setiawati et al. showed that the slope of TIC as a parameter could improve the diagnostic accuracy of osteosarcoma subtypes (36). These studies indicated the potential of Sp in breast lesion classification, but in this study, Sp was not an independent predictor in the multivariable analysis. This could be partially explained by the small sample size of this study.

Interestingly, the ERp was lower in grade III lesions compared with grade I–II lesions, and no relevant literature could be found to explain the result or for comparison. This unexpected finding warrants further investigation.

This study has several limitations. First, its retrospective, single-center design and modest sample size limit generalizability, and our findings require external validation. Second, the ROIs were manually delineated, and observer bias is inevitable. Future work with automated segmentation could mitigate this. Third, the age distribution was imbalanced in this study, and the patients in the malignant group were older. This represents a potential confounding factor.

## 5 Conclusion

This study suggests that the DCE-T1WI parameters are different in benign and malignant breast lesions with type II DCE TIC. Tp was independently associated with malignant lesions and has potential in clinical practice. A nomogram provides a visualization tool for breast lesion classification.

## Data Availability

The original contributions presented in the study are included in the article/supplementary material. Further inquiries can be directed to the corresponding author.
